# Risk of Safety Events in Vitiligo Patients: A Retrospective Real‐World Data Study in the US

**DOI:** 10.1111/1346-8138.70256

**Published:** 2026-04-06

**Authors:** Kennedy Cook, Nada M. Elbuluk, Roni Adiri, Alexandre Lejeune, Thomas Edwards, Milena A. Gianfrancesco, Scott P. Kelly, Tatjana Lukic, Edward Nagy, Samantha K. Kurosky, Lynne Napatalung, Iltefat Hamzavi

**Affiliations:** ^1^ Pfizer Inc New York New York USA; ^2^ Department of Dermatology Keck School of Medicine, University of Southern California Los Angeles California USA; ^3^ Pfizer Pharmaceuticals Israel Ltd. Herzliya Israel; ^4^ Pfizer Inc Paris France; ^5^ Panalgo, LLC Westland Michigan USA; ^6^ Pfizer Inc Collegeville Pennsylvania USA; ^7^ Department of Dermatology Icahn School of Medicine at Mount Sinai New York New York USA; ^8^ Henry Ford Health System Department of Dermatology Detroit Michigan USA

**Keywords:** electronic health records, epidemiology, observational study, pigmentation disorders, vitiligo

## Abstract

Vitiligo is a chronic autoimmune depigmenting disease characterized by loss of pigment in the skin, hair, or both. As treatment options evolve, particularly with the emergence of oral Janus kinase inhibitors and dual Janus kinase 3/tyrosine kinase expressed in hepatocellular carcinoma family kinase inhibitor, it is essential to assess population‐level safety events in patients with vitiligo. This retrospective cohort study examined patients aged ≥ 12 years with vitiligo in the United States, comparing them with age‐, sex‐, and race‐matched controls to evaluate patient demographics, baseline comorbidities, medication histories, vitiligo prevalence and incidence across demographic groups, and incidence rates of safety events following vitiligo diagnosis. This study used records dated 1 January 2016 to 30 September 2023 from the Optum Market Clarity United States Electronic Health Record database. This study included 15 047 patients with vitiligo and 75 231 matched controls. In both cohorts, the median age was 51 years and 56.3% of patients were female. Compared with controls, patients with vitiligo had a higher baseline proportion of autoimmune and inflammatory diseases (e.g., autoimmune thyroiditis, psoriasis, alopecia areata, and atopic dermatitis), infections, malignancies, hypothyroidism, allergic rhinitis, and hearing loss; they were also more likely to have used treatments for dermatologic conditions and immunosuppressive medications. Post diagnosis, higher incidence rates of autoimmune and inflammatory conditions (e.g., alopecia areata, systemic sclerosis, pernicious anemia, autoimmune thyroiditis, and psoriasis), infections, hearing‐related events, and skin cancer were observed in the vitiligo cohort compared with the non‐vitiligo cohort. Furthermore, vitiligo incidence and prevalence rates were numerically higher in Asian and Hispanic populations than in other racial and ethnic groups. However, the observed results should be interpreted with caution considering the limitations of the dataset. These findings provide valuable insight into the epidemiology of and safety considerations for patients with vitiligo in the United States, informing future clinical and therapeutic strategies.

## 1 Introduction

Vitiligo is a chronic autoimmune depigmenting disease characterized by loss of pigment in the skin, hair, or both due to melanocyte destruction [1]. The estimated lifetime prevalence of vitiligo diagnosed by a physician varies globally, with 0.36% (95% credible interval [CrI], 0.24–0.54) in the general population, 0.67% (CrI, 0.43–1.07) among adults, and 0.24% (CrI, 0.16–0.37) among children [2]. In the United States (US), the lifetime prevalence of physician‐diagnosed vitiligo is estimated to be 0.50% (CrI, 0.34–0.75) among adults and 0.18% (CrI, 0.12–0.28) among children [2]. There are two major subtypes of vitiligo. One subtype is segmental vitiligo, with unilateral presentation confined to the dermatome [3, 4]; it comprises 5% to 16% of vitiligo cases and is rarely associated with systemic autoimmune disorders (the neuronal hypothesis and somatic mosaicism are considered the major factors in its pathogenesis, rather than autoimmunity [5]). The more common form is nonsegmental vitiligo; with wide bilateral distribution and autoimmune etiology, it comprises 85% to 90% of cases [1, 6].

Vitiligo impacts patients beyond its effects on skin pigmentation. Vitiligo is known to significantly affect patients' health‐related quality of life, largely due to the psychosocial burden associated with social stigma [1]. Compared with healthy controls, individuals with vitiligo have higher rates of anxiety‐related and depressive disorders, along with other psychosocial comorbidities [1, 7, 8]. Vitiligo is also associated with an increased risk of other autoimmune and inflammatory conditions, including thyroid disorders, alopecia areata (AA), type 1 diabetes, and atopic dermatitis [9, 10, 11, 12, 13]. In the US, a survey reported that patients with vitiligo were 2.65 times more likely to have an autoimmune or autoinflammatory comorbidity such as psoriasis, rheumatoid arthritis, thyroid disease, or multiple sclerosis than individuals without vitiligo [10].

Treatments for vitiligo typically include topical and systemic corticosteroids, topical calcineurin inhibitors, phototherapy, and surgical procedures, among others [14, 15]. Ruxolitinib cream, a Janus kinase (JAK) 1/2 inhibitor, was approved by the US Food and Drug Administration in 2022 and the European Medicines Agency in 2023 for the treatment of nonsegmental vitiligo in adults and children ≥ 12 years of age for application to affected areas of up to 10% of the body's surface area [16]. Advanced systemic treatments are currently under investigation for vitiligo, including ritlecitinib, an oral selective JAK3/tyrosine kinase expressed in hepatocellular carcinoma (TEC) family kinase inhibitor, and upadacitinib and povorcitinib, both JAK1 inhibitors [17, 18, 19, 20].

Some JAK inhibitors have been associated with an increased risk of certain adverse events, including infections, malignancies, and cardiovascular events [21, 22]. As new medication classes, such as oral kinase inhibitors, are developed for systemic treatment of vitiligo, it is essential to assess the current population‐level risk for specific safety events potentially associated with these therapies [21, 22]. Such an evaluation can contextualize adverse events observed in clinical trials, guide real‐world treatment decisions by accounting for patients' baseline risk profiles, and support more informed benefit–risk assessments for regulators, healthcare providers, and patients.

Although vitiligo epidemiology has been examined in prior US studies, most have relied on self‐reported surveys or administrative claims data, and published US incidence estimates are scarce. The present study leverages a large national electronic health records (EHRs) database to provide real‐world estimates of diagnosed vitiligo incidence and prevalence, as well as incidence rates (IRs) of selected safety events of interest, across a diverse population, thereby addressing important gaps in real‐world disease burden. This study compared a cohort of patients with vitiligo with an age‐, race‐, and sex‐matched cohort of patients without vitiligo in the US. The objectives were to (1) describe baseline characteristics, comorbidities, and medication histories; (2) estimate and compare the IRs of selected safety events of interest (including autoimmune conditions, inflammatory conditions, infections, malignancies, psychosocial impacts, major adverse cardiac events [MACE], and venous thromboembolism); and (3) estimate the prevalence, incidence, and cumulative incidence of vitiligo stratified by age, ethnicity, race, and sex.

## 2 Methods

### 2.1 Data Source

**FIGURE 1:**
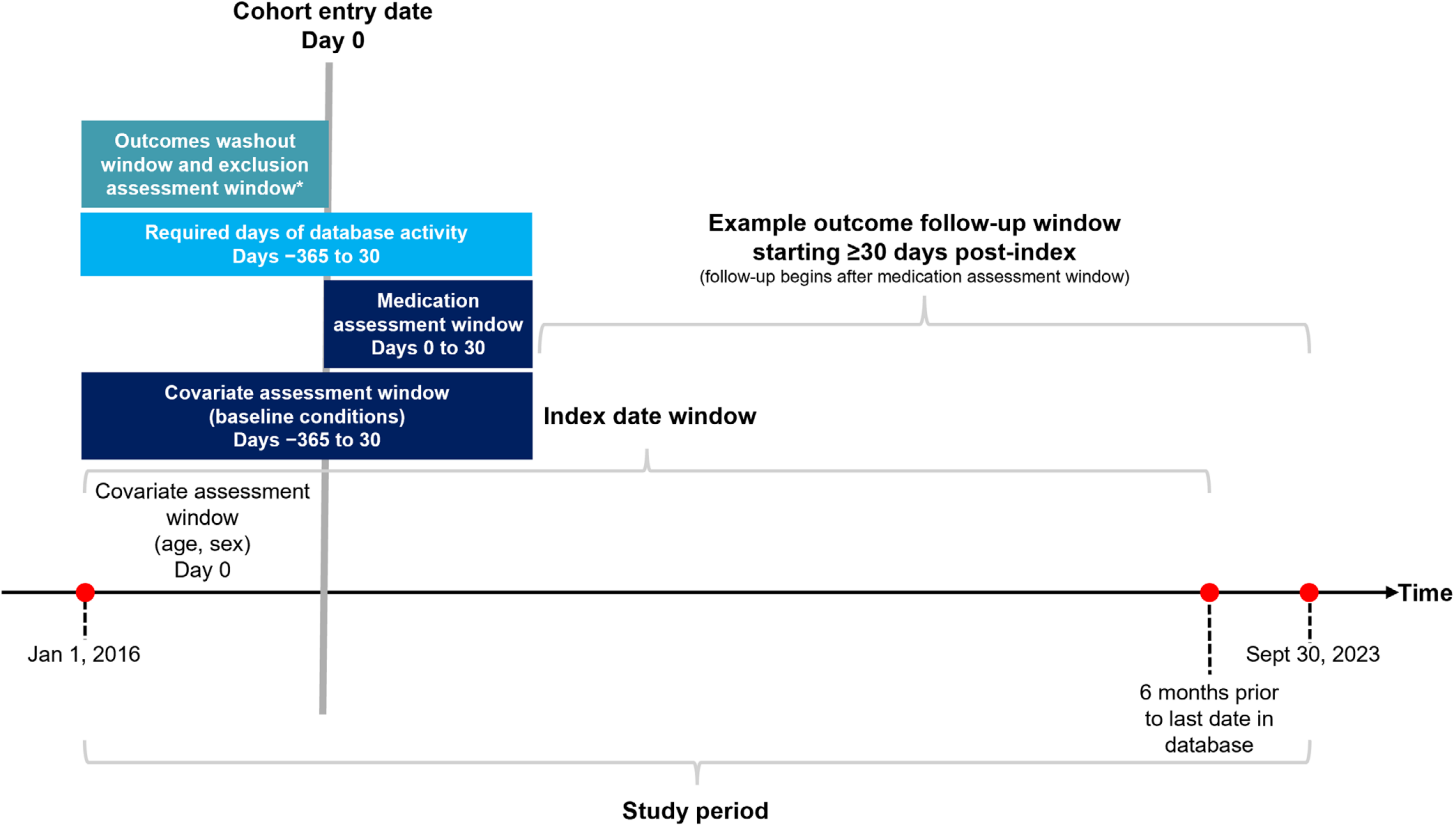
Study design. *Washout periods and assessment windows for each variable are shown in Table S1.

This population‐based retrospective cohort study used the Optum Market Clarity US EHR database, encompassing data from 1 January 2016 to 30 September 2023 (Figure 1). The database includes over 700 hospitals and 7000 clinics, representing more than 100 million patients, and integrates clinical, claims, and other medical administrative data. All records were deidentified in accordance with the Health Insurance Portability and Accountability Act; therefore, neither obtaining informed consent from patients nor approval from an institutional review board was required.

### 2.2 Patient Population

The study population consisted of patients diagnosed with vitiligo (vitiligo cohort) and a matched cohort of patients without vitiligo (non‐vitiligo cohort). Inclusion criteria required patients to be at least 12 years of age at the index date, have at least 365 days of database activity prior to the index date, and have a minimum of 30 days of activity following the index date (Figure 1). Patients with missing data for the date of birth or sex during the 365‐day pre‐index period were excluded.

Patients in the vitiligo cohort were required to have a qualifying vitiligo diagnosis during the study period, defined as either a diagnosis from a dermatologist or the second of two vitiligo diagnoses made at least 30 days apart by a non‐dermatologist clinician. The index date for vitiligo patients was the date of the first qualifying vitiligo diagnosis recorded during the study period, which may not reflect the date of vitiligo onset.

The non‐vitiligo cohort included patients with no recorded vitiligo diagnosis at any time and was matched to the vitiligo cohort at a ratio of up to 5:1 based on exact matching for age, sex, race, and index year‐month. The index date for non‐vitiligo patients was the nearest date of healthcare activity within 31 days of the matched vitiligo patient's index date. These patients were also required to have documented medical activity within 31 days of their index date.

### 2.3 Study Variables

Study variables, medication classes, and events are listed in Table S1. Demographic variables recorded included age, sex, race, ethnicity, body mass index category, smoking status, US census region, payor type, index year, and number of dermatologist visits. Baseline comorbidities, history of surgical procedures, and prescription medication history were captured if they occurred within the baseline period through 30 days after the index date. The baseline period was defined as the 365 days immediately prior to the index date within the dataset and does not necessarily correspond to the period before disease onset. Baseline comorbidities assessed included autoimmune and inflammatory conditions, infections, psychiatric conditions, malignancies, cardiovascular conditions, and other conditions (allergic rhinitis, asthma, chronic obstructive pulmonary disease, diabetes, hearing loss, hypothyroidism, and type 2 diabetes). Crude IRs for safety events of interest were estimated for events occurring ≥ 30 days post‐index date with pre‐index washout periods for certain events. Safety events included autoimmune and inflammatory, infection, psychiatric, malignancy, cardiovascular‐related, and other outcomes, as well as death.

All diagnosis data and surgical procedure data were obtained from the structured data derived from the *International Classification of Diseases (ICD) 9 or 10*, *Clinical Modification Procedure Classification System (PCS)* (*ICD‐9‐PCS/ICD‐10‐PCS*), or current procedural terminology codes where applicable. All drug treatment data were derived from written prescriptions, medication administration, and procedure tables when appropriate through the National Drug Center, current procedural terminology, and Healthcare Common Procedure Coding System codes where applicable. Lists of diagnosis, procedure, and prescription codes are provided in File S1.

### 2.4 Statistical Analysis

Descriptive statistics were used to summarize baseline characteristics and comorbidities, history of surgical procedures, prescription medication use, and IRs of safety events of interest for the study cohorts; no inferential statistical testing was conducted. Categorical variables were reported as frequencies and percentages, while continuous variables were summarized using means and standard deviations (SDs) or medians with interquartile ranges (IQRs) as appropriate.

The incidence of diagnosed vitiligo was estimated and stratified by age, ethnicity, race, and sex. Incidence rates were calculated as the number of incident vitiligo cases divided by patient‐years at risk, with 95% confidence intervals (CIs). Incident cases were defined as individuals with no prior diagnosis codes for vitiligo prior to the index date. Patients with missing race were not excluded from this analysis. Cumulative incidence was calculated using a Kaplan–Meier approach, with a modification to account for death as a competing event rather than censoring. Specifically, instead of assuming all individuals will eventually be diagnosed with vitiligo and censoring those who died, death was treated as a competing event and a person's risk for developing vitiligo was set to 0 when they died [23]. This approach prevented overestimation of cumulative incidence that can occur in traditional Kaplan–Meier analyses, which assume all individuals remain at risk unless censored. This approach excluded non–at‐risk person‐time from the denominator. Cumulative incidence estimates were stratified by age, ethnicity, race, and sex. Vitiligo prevalence was calculated as the number of total cases divided by the total number of patients in the dataset, with 95% CI, and was also stratified by age, ethnicity, race, and sex. Descriptive statistics were used to summarize all stratified incidence and prevalence estimates, and no inferential statistical testing was performed. All analyses were performed using the Instant Health Data software (Panalgo, Boston, MA) and R, version 3.2.1 (R Foundation for Statistical Computing, Vienna, Austria).

Crude IRs of events of interest were estimated, with follow‐up beginning 30 days after the index date and ending on the date of the first‐recorded key disease event, death, or end of the study period, whichever occurred first. Patients who experienced an event were censored for that specific outcome but remained eligible for the evaluation of other events or categories. The washout period for each variable is shown in Table S1; acute events had no washout period, while other conditions had a washout for the entire database availability pre‐index [−∞, index date −1]. For chronic conditions (malignancy, any MACE, deep vein thromboembolism events, pulmonary embolism events, and arterial embolism), IRs were computed in subcohorts that excluded those with evidence of having had the event at any time before the index date. For MACE, this meant that the subcohort in which MACE IRs were estimated consisted exclusively of patients with no prior evidence of any one of the MACE events in their EHR. Only the first record of a given disease event was considered when calculating IRs.

Pfizer's generative artificial intelligence–assisted technology was used in the production of the text of this manuscript. After using this tool, the authors reviewed and edited the content and take full responsibility for the final content of the publication.

## 3 Results

### 3.1 Patient Demographics

A total of 15 047 patients with vitiligo and 75 231 non‐vitiligo matched controls were included in this study. Given the matching strategy used, the cohorts had a comparable median age (51.0 years), sex distribution (56.3% female), and racial composition (68.2% White, 13.0% Black, and 5.6% Asian; Table 1). The majority of patients in both cohorts were non‐Hispanic, although this proportion was slightly lower in the vitiligo cohort (76.9% vitiligo and 81.6% non‐vitiligo). Geographic distribution differed between cohorts: the vitiligo group compared with the non‐vitiligo group included a lower proportion of patients from the Midwest (45.7% vs. 62.4%) and South (12.9% vs. 17.0%), and a higher proportion from the Northeast (28.2% vs. 13.2%) and West (8.3% vs. 3.6%). Body mass index group, index year, and payor type were also generally comparable between cohorts.

**TABLE 1:** Patient characteristics among patients with and without vitiligo.

	Vitiligo matched cohort (*n* = 15 047)	Non‐vitiligo matched cohort (*n* = 75 231)
Age at index, years		
Mean (SD)	48.9 (18.9)	48.9 (18.9)
Median (IQR)	51 (35–64)	51 (35–64)
Min‐max	12–88	12–88
Sex, *n* (%)		
Female	8469 (56.3)	42 344 (56.3)
Male	6578 (43.7)	32 887 (43.7)
Race, *n* (%)		
Asian	843 (5.6)	4211 (5.6)
Black	1960 (13.0)	9800 (13.0)
White	10 257 (68.2)	51 285 (68.2)
Unknown	1987 (13.2)	9935 (13.2)
Ethnicity, *n* (%)		
Hispanic	1706 (11.3)	6186 (8.2)
Not Hispanic	11 564 (76.9)	61 422 (81.6)
Unknown	1777 (11.8)	7623 (10.1)
Smoking status, *n* (%)		
Current	742 (4.9)	7739 (10.3)
Former	2481 (16.5)	13 864 (18.4)
Never	6900 (45.9)	22 533 (30.0)
Unknown	4924 (32.7)	31 095 (41.3)
Body mass index group, *n* (%)		
< 18.5	280 (1.9)	1505 (2)
18.5–24.9	3456 (23.0)	14 591 (19.4)
25–29.9	3879 (25.8)	17 245 (22.9)
30–34.9	2296 (15.3)	13 106 (17.4)
35–39.9	996 (6.6)	6967 (9.3)
40+	680 (4.5)	5800 (7.7)
Unknown	3460 (23.0)	16 017 (21.3)
Index year, *n* (%)		
2017	3808 (25.3)	19 039 (25.3)
2018	3040 (20.2)	15 199 (20.2)
2019	3014 (20.0)	15 070 (20.0)
2020	1899 (12.6)	9494 (12.6)
2021	1663 (11.1)	8315 (11.1)
2022	1342 (8.9)	6709 (8.9)
2023	281 (1.9)	1405 (1.9)
Census region, *n* (%)		
Midwest	6876 (45.7)	46 965 (62.4)
Northeast	4245 (28.2)	9930 (13.2)
South	1933 (12.9)	12 763 (17.0)
West	1247 (8.3)	2695 (3.6)
Unknown	746 (5.0)	2878 (3.8)
Payor type, *n* (%)		
Commercial	8263 (54.9)	38 974 (51.8)
Medicaid	1204 (8.0)	7639 (10.2)
Medicare	2509 (16.7)	14 192 (18.9)
Uninsured	524 (3.5)	2411 (3.2)
Other	232 (1.5)	995 (1.3)
Unknown	2315 (15.4)	11 020 (14.7)

### 3.2 Baseline Comorbidities and History of Prescription Medication Use and Surgical Treatments

A numerically greater proportion of patients in the vitiligo cohort had baseline autoimmune and inflammatory comorbidities than did those in the non‐vitiligo cohort. These comorbidities included autoimmune thyroiditis, Hashimoto's thyroiditis, psoriasis, AA, atopic dermatitis, type 1 diabetes, Graves' disease, systemic lupus erythematosus, celiac disease, psoriatic arthritis, ulcerative colitis, myasthenia gravis, multiple sclerosis, and rheumatoid arthritis (Table 2). Infectious events were also more common in the vitiligo cohort at baseline, including herpes simplex, serious infection requiring hospitalization, herpes zoster, and human immunodeficiency virus (HIV), as well as hepatitis C (Table 2). Certain malignancies were more common in the vitiligo cohort than the non‐vitiligo cohort at baseline. These included melanoma, non‐melanoma skin cancer (NMSC), and primary malignancy, as well as Epstein–Barr–related lymphoma, Epstein–Barr–related leukemia, and lymphoma (Table 2).

**TABLE 2:** Proportion of patients with baseline comorbidities in the vitiligo matched and non‐vitiligo matched cohorts.

Category, *n* (%)	Vitiligo matched cohort (*n* = 15 047)	Non‐vitiligo matched cohort (*n* = 75 231)
Autoimmune and inflammatory		
Autoimmune thyroiditis	535 (3.6)	432 (0.6)
Hashimoto's thyroiditis	535 (3.6)	432 (0.6)
Psoriasis	636 (4.2)	726 (1.0)
Alopecia areata	190 (1.3)	46 (0.1)
Atopic dermatitis	324 (2.2)	376 (0.5)
Type 1 diabetes	374 (2.5)	755 (1.0)
Graves' disease	164 (1.1)	211 (0.3)
Systemic lupus erythematosus	132 (0.9)	284 (0.4)
Celiac disease	76 (0.5)	134 (0.2)
Psoriatic arthritis	91 (0.6)	205 (0.3)
Ulcerative colitis	116 (0.8)	292 (0.4)
Myasthenia gravis	27 (0.2)	48 (0.1)
Multiple sclerosis	50 (0.3)	426 (0.6)
Rheumatoid arthritis	94 (0.6)	334 (0.4)
Crohn's disease	82 (0.5)	370 (0.5)
Infection		
Herpes simplex	329 (2.2)	930 (1.2)
Serious infection requiring hospitalization	547 (3.6)	2053 (2.7)
Herpes zoster	147 (1.0)	462 (0.6)
HIV	70 (0.5)	185 (0.3)
Hepatitis C	123 (0.8)	465 (0.6)
Hepatitis B	33 (0.2)	158 (0.2)
Malignancy		
Melanoma	168 (1.1)	113 (0.2)
Basal cell carcinoma	174 (1.2)	335 (0.5)
NMSC	119 (0.8)	205 (0.3)
Squamous cell carcinoma	87 (0.6)	130 (0.2)
Primary malignancy	1004 (6.7)	3766 (5.0)
EBV‐related lymphoma	83 (0.6)	289 (0.4)
EBV‐related leukemia	9 (0.1)	15 (0.0)
Lymphoma	78 (0.5)	287 (0.4)
Cervical cancer in situ	14 (0.1)	46 (0.1)
EBV‐related lymphoproliferative disorder	40 (0.3)	166 (0.2)
Breast cancer	185 (1.2)	960 (1.3)
Leukemia	47 (0.3)	221 (0.3)
Psychiatric‐related		
Substance use disorder	333 (2.2)	2535 (3.4)
Anxiety	2350 (15.6)	13 259 (17.6)
Mood disorder	344 (2.3)	2336 (3.1)
Suicidal Ideation	57 (0.4)	553 (0.7)
Personality disorder	25 (0.2)	239 (0.3)
Cardiovascular‐related		
Hypertension	4206 (28.0)	24 431 (32.5)
CABG or PCI	162 (1.1)	1370 (1.8)
VTE or ATE	349 (2.3)	1962 (2.6)
Ischemic stroke	199 (1.3)	1158 (1.5)
Arterial thromboembolism	190 (1.3)	1082 (1.4)
Hyperlipidemia	4529 (30.1)	22 137 (29.4)
Pulmonary embolism	86 (0.6)	505 (0.7)
Unstable angina	37 (0.3)	229 (0.3)
VTE	183 (1.2)	980 (1.3)
Acute myocardial infarction	141 (0.9)	668 (0.9)
Dyslipidemia	3848 (25.6)	19 086 (25.4)
Deep vein thrombosis	129 (0.9)	622 (0.8)
Hemorrhagic stoke	43 (0.3)	203 (0.3)
Other		
Hypothyroidism	2612 (17.4)	6668 (8.9)
Allergic rhinitis	1293 (8.6)	4692 (6.2)
Hearing loss	520 (3.5)	1571 (2.1)
Type 2 diabetes	1699 (11.3)	10 442 (13.9)
COPD	471 (3.1)	3523 (4.7)
Diabetes	1877 (12.5)	10 820 (14.4)
Asthma	1291 (8.6)	6478 (8.6)

Abbreviations: ATE, arterial thromboembolism; CABG, coronary artery bypass graft; COPD, chronic obstructive pulmonary disease; EBV, Epstein–Barr virus; NMSC, non‐melanoma skin cancer; PCI, percutaneous coronary intervention; VTE, venous thromboembolism.

Psychiatric comorbidities, including substance use disorder, anxiety, mood disorder, suicidal ideation, and personality disorder, were less common in the vitiligo cohort than the non‐vitiligo cohort at baseline. Similarly, certain cardiovascular‐related comorbidities, such as hypertension, coronary artery bypass graft or percutaneous coronary intervention, venous thromboembolism or arterial thromboembolism, and ischemic stroke, were also less common in the vitiligo cohort vs the non‐vitiligo cohort at baseline. Other cardiovascular‐related comorbidities, including arterial thromboembolism, hyperlipidemia, pulmonary embolism, unstable angina, venous thromboembolism, acute myocardial infarction, dyslipidemia, deep vein thromboembolism, and hemorrhagic stroke, did not differ substantially between the two groups (Table 2). Among other comorbidities, hypothyroidism, allergic rhinitis, and hearing loss were more common in patients with vitiligo at baseline than in patients without vitiligo (Table 2).

Having a history of prescription medication use and surgical treatments was more common at baseline among patients with vitiligo than those without (Table 3). Notable differences among patients with and without vitiligo included a higher use of laser treatments (9.1% vs. 2.1%), phototherapy (3.3% vs. 0.0%), and surgical treatments and camouflage (3.2% vs. 0.8%), as well as greater use of systemic corticosteroids (betamethasone, hydrocortisone, cortisone, prednisone, prednisolone, or triamcinolone; 36.7% vs. 19.8%), systemic steroids (including glucocorticoids; 44.4% vs. 28.1%), systemic immune suppressants (cyclosporine A, tacrolimus, azathioprine, methotrexate, sulfasalazine, mycophenolate mofetil, everolimus, or ibrutinib; 21.3% vs. 1.3%), topical calcineurin inhibitors (24.0% vs. 0.3%), and topical corticosteroids (25.9% vs. 7.0%). Patients in the vitiligo cohort had substantially more dermatologist visits at baseline than the patients in the non‐vitiligo cohort (mean [SD], 1.8 [8.9] vs. 0.2 [1.4]).

**TABLE 3:** History of baseline prescription medication use, surgical treatments, and dermatologists visits among patients with and without vitiligo.

*n* (%)	Vitiligo matched cohort (*n* = 15 047)	Non‐vitiligo matched cohort (*n* = 75 231)
Antidiabetic agents	1706 (11.3)	10 299 (13.7)
Antihypertensive agents	4136 (27.5)	26 432 (35.1)
Dermatology drugs	205 (1.4)	506 (0.7)
Dermatologist visit	1.8 (8.9)	0.2 (1.4)
Hormone replacement therapy	128 (0.9)	479 (0.6)
Hysterectomy	182 (1.2)	1143 (1.5)
Immunosuppressants	2557 (17.0)	12 457 (16.6)
Intralesional corticosteroids	3626 (24.1)	17 307 (23.0)
JAK inhibitors (oral and topical)	49 (0.3)	70 (0.1)
Laser	1373 (9.1)	1570 (2.1)
Lipid modifying agents	2625 (17.5)	15 089 (20.1)
Non‐B cell lymphocyte depleting agents	14 (0.1)	49 (0.1)
Oophorectomy	66 (0.4)	436 (0.6)
Oral contraceptives	693 (4.6)	3982 (5.3)
Other immunomodulatory agents	209 (1.4)	510 (0.7)
Phototherapy	500 (3.3)	15 (0.0)
Salpingectomy	63 (0.4)	411 (0.6)
Surgical treatments and camouflage	475 (3.2)	586 (0.8)
Systemic corticosteroids	5514 (36.7)	14 910 (19.8)
Systemic steroids	6681 (44.4)	21 158 (28.1)
Systemic immune suppressants	3204 (21.3)	995 (1.3)
Topical calcineurin inhibitors	3610 (24.0)	198 (0.3)
Topical corticosteroids	3896 (25.9)	5263 (7.0)
Topical vitamin D3	159 (1.1)	90 (0.1)
Zoster vaccine live	52 (0.4)	237 (0.3)
Zoster vaccine recombinant	230 (1.5)	681 (0.9)
Photochemotherapy (PUVA)	5 (0.0)	1 (0.0)

Abbreviations: JAK, Janus kinase; PUVA, psoralen plus ultraviolet A.

### 3.3 Prevalence and Incidence of Vitiligo

**FIGURE 2:**
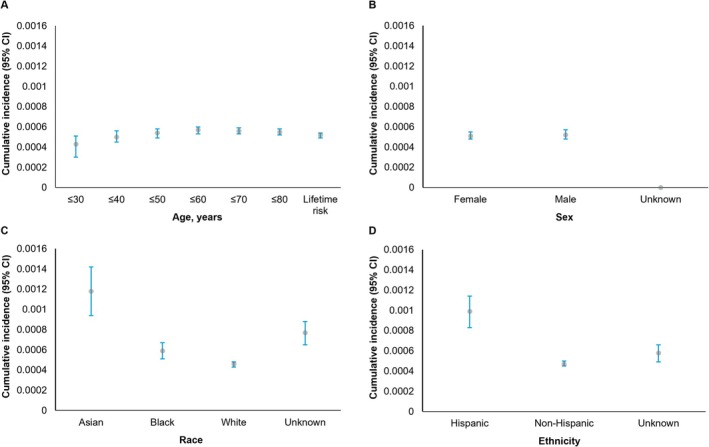
Cumulative incidence of vitiligo stratified by age, sex, race, and ethnicity.

The prevalence and incidence of vitiligo were estimated and stratified by age, sex, race, ethnicity, and overall (Table 4). Asian populations had a numerically higher prevalence (1.19; 95% CI, 1.05–1.35) and incidence (IR, 0.19; 95% CI, 0.16–0.22) of vitiligo than other racial groups (Black, White, unknown) with vitiligo. Similarly, Hispanic populations demonstrated a numerically higher prevalence (0.87; 95% CI, 0.79–0.95) and incidence (IR, 0.15; 95% CI, 0.13–0.17) of vitiligo than non‐Hispanic populations. Observed prevalence and incidence rates of vitiligo were generally comparable across age and sex stratifications. Cumulative incidence of vitiligo was also numerically higher among Asian (0.00118; 95% CI, 0.00094–0.00142) and Hispanic populations (0.00099; 95% CI, 0.00083–0.00114) relative to other racial and ethnic groups with vitiligo (Figure 2). The observed cumulative incidence of vitiligo was generally similar across age and sex stratifications (Figure 2).

**TABLE 4:** Prevalence and incidence of vitiligo by stratification.

Category	Prevalence per 1000 patients (95% CI)	IR per 1000 PY (95% CI)
Age		
12–17 years	0.60 (0.51–0.64)	0.07 (0.06–0.08)
18–50 years	0.46 (0.44–0.49)	0.08 (0.08–0.08)
51+ years	0.54 (0.52–0.57)	0.08 (0.08–0.09)
Pediatric (< 18 years of age)	0.57 (0.51–0.64)	0.07 (0.06–0.08)
Adult (> 18 years of age)	0.50 (0.49–0.52)	0.08 (0.08–0.08)
Sex		
Female	0.50 (0.48–0.52)	0.08 (0.08–0.08)
Male	0.52 (0.49–0.54)	0.08 (0.08–0.09)
Unknown	0.08 (0.00–0.47)	0.00 (0.00–0.14)
Race		
Asian	1.19 (1.05–1.35)	0.19 (0.16–0.22)
Black	0.54 (0.50–0.59)	0.09 (0.08–0.10)
Caucasian	0.48 (0.46–0.50)	0.07 (0.07–0.08)
Unknown	0.51 (0.47–0.55)	0.10 (0.09–0.11)
Ethnicity		
Hispanic	0.87 (0.79–0.95)	0.15 (0.13–0.17)
Not Hispanic	0.51 (0.49–0.53)	0.08 (0.07–0.08)
Unknown	0.37 (0.34–0.41)	0.07 (0.06–0.08)
Overall, crude	0.51 (0.49–0.52)	0.08 (0.08–0.08)

Abbreviations: IR, incidence rate; PY, patient years.

### 3.4 Incidence of Safety Events of Interest Among Patients With and Without Vitiligo

IRs for safety events were numerically greater for several conditions in the vitiligo cohort compared with the non‐vitiligo cohort (Table 5). Patients with vitiligo had higher IRs of certain autoimmune and inflammatory events than patients without vitiligo, including AA (IR per 1000 PY [95% CI], 4.52 [3.9–5.25] vs. 0.34 [0.26–0.44]), autoimmune blistering disease (0.73 [0.51–1.05] vs. 0.26 [0.19–0.35]), autoimmune thyroiditis (5.90 [5.16–6.74] vs. 1.46 [1.28–1.66]), bullous pemphigoid (0.73 [0.51–1.05] vs. 0.24 [0.18–0.33]), myasthenia gravis (0.23 [0.12–0.44] vs. 0.14 [0.09–0.21]), pernicious anemia (1.07 [0.79–1.45] vs. 0.26 [0.19–0.35]), psoriasis (4.36 [3.73–5.1] vs. 2.06 [1.85–2.3]), Sjogren's syndrome (1.89 [1.5–2.38] vs. 0.86 [0.73–1.02]), systemic lupus erythematosus (1.1 [0.82–1.49] vs. 0.50 [0.40–0.62]), systemic sclerosis (0.57 [0.38–0.87] vs. 0.11 [0.07–0.17]), and type 1 diabetes (1.74 [1.37–2.22] vs. 1.5 [1.32–1.7]). For infection‐related events, higher IRs were observed in the vitiligo cohort compared with the non‐vitiligo cohort for herpes simplex (IR per 1000 PY [95% CI], 5.83 [5.1–6.67] vs. 4.75 [4.42–5.1]), herpes zoster (5.14 [4.46–5.92] vs. 4.09 [3.79–4.42]), and opportunistic infections (2.76 [2.28–3.34] vs. 1.95 [1.75–2.18]).

**TABLE 5:** IRs for safety events of interest in the vitiligo and non‐vitiligo matched cohorts.

IR per 1000 PY (95% CI)	Vitiligo matched cohort (*n* = 15 047)	Non‐vitiligo matched cohort (*n* = 75 231)
Autoimmune and inflammatory events		
Alopecia areata	4.52 (3.9–5.25)	0.34 (0.26–0.44)
Autoimmune blistering diseases	0.73 (0.51–1.05)	0.26 (0.19–0.35)
Autoimmune thyroiditis	5.90 (5.16–6.74)	1.46 (1.28–1.66)
Bullous pemphigoid	0.73 (0.51–1.05)	0.24 (0.18–0.33)
Epidermolysis bullosa	0.00 (0.00–0.10)	0.01 (0.00–0.04)
Myasthenia gravis	0.23 (0.12–0.44)	0.14 (0.09–0.21)
Pemphigus vulgaris	0.05 (0.02–0.19)	0.03 (0.01–0.07)
Pernicious anemia	1.07 (0.79–1.45)	0.26 (0.19–0.35)
Psoriasis	4.36 (3.73–5.1)	2.06 (1.85–2.3)
Rheumatoid arthritis	0.94 (0.68–1.31)	1.09 (0.94–1.26)
Sjogren's syndrome	1.89 (1.5–2.38)	0.86 (0.73–1.02)
Systemic lupus erythematosus	1.1 (0.82–1.49)	0.50 (0.40–0.62)
Systemic sclerosis	0.57 (0.38–0.87)	0.11 (0.07–0.17)
Type 1 diabetes	1.74 (1.37–2.22)	1.5 (1.32–1.7)
Infection‐related events		
Herpes simplex	5.83 (5.1–6.67)	4.75 (4.42–5.1)
Herpes zoster	5.14 (4.46–5.92)	4.09 (3.79–4.42)
Opportunistic infection	2.76 (2.28–3.34)	1.95 (1.75–2.18)
Serious infection	215.8 (210.23–221.52)	216.95 (214.27–219.66)
Psychosocial events		
Depression	26.63 (24.88–28.52)	35.25 (34.2–36.33)
Psychiatric conditions	61.85 (58.68–65.21)	78.23 (76.3–80.22)
Suicidal ideation	2.68 (2.21–3.25)	4.87 (4.54–5.22)
Malignancy events		
Melanoma	0.74 (0.49–1.06)	0.65 (0.53–0.78)
NMSC	3.96 (3.38–4.64)	2.04 (1.83–2.27)
Primary malignancy	33.85 (32–35.81)	29.45 (28.61–30.31)
Cardiovascular‐related events		
Acute myocardial infarction	6.49 (5.73–7.35)	7.65 (7.23–8.08)
Arterial thrombosis	9.34 (8.42–10.37)	9.93 (9.45–10.43)
CABG or PCI	6.51 (5.75–7.37)	10.12 (9.64–10.62)
Cardiovascular death	177.39 (153.21–205.46)	155.92 (145.91–166.62)
DVT	5.15 (4.48–5.92)	5.81 (5.45–6.19)
DVT or PE	7.46 (6.64–8.37)	8.83 (8.38–9.29)
DVT, PE, or arterial thrombosis	15.82 (14.59–17.14)	17.99 (17.34–18.66)
Heart failure hospitalization	7.80 (6.97–8.74)	10.52 (10.03–11.03)
Hemorrhagic stroke	1.95 (1.55–2.44)	1.96 (1.76–2.19)
Ischemic stroke	10.07 (9.11–11.13)	10.58 (10.09–11.09)
MACE	26.11 (24.51–27.82)	31.88 (31–32.78)
Pulmonary embolism	3.52 (2.98–4.17)	4.37 (4.07–4.71)
Unstable angina	1.02 (0.74–1.39)	1.60 (1.41–1.80)
Venous thromboembolism	9.54 (8.61–10.58)	10.51 (10.02–11.02)
Other events		
Diabetes	12.60 (11.43–13.88)	16.15 (15.48–16.85)
Hearing loss	13.99 (12.81–15.29)	10.15 (9.65–10.66)
Paresthesia or dysesthesia	24 (22.39–25.72)	21.42 (20.67–22.19)
Peripheral neuropathy	19.95 (18.49–21.52)	20.11 (19.38–20.86)
Sensorineural hearing loss	8.75 (7.85–9.77)	5.13 (4.79–5.49)
Type 2 diabetes	13.33 (12.13–14.64)	16.33 (15.66–17.03)
Death	8.57 (7.69–9.54)	11.38 (10.88–11.91)

Abbreviations: CABG, coronary artery bypass graft; DVT, deep vein thrombosis; IR, incidence rate; MACE, major adverse cardiovascular events; NMSC, non‐melanoma skin cancer; PCI, percutaneous coronary intervention; PE, pulmonary embolism.

Observed IRs for psychiatric conditions, cardiovascular events, and death were lower in patients with vitiligo vs. patients without vitiligo (Table 5). Patients with vitiligo had numerically higher IRs of malignancy events vs. those without, including melanoma (IR per 1000 PY [95% CI], 0.74 [0.49–1.06] vs. 0.65 [0.53–0.78]), NMSC (3.96 [3.38–4.64] vs. 2.04 [1.83–2.27]), and primary malignancy (33.85 [32–35.81] vs. 29.45 [28.61–30.31]). The IRs of hearing‐related events were also numerically higher in the vitiligo cohort than in the non‐vitiligo cohort, including hearing loss (IR per 1000 PY [95% CI], 13.99 [12.81–15.29] vs. 10.15 [9.65–10.66]), paresthesia or dysesthesia (24 [22.39–25.72] vs. 21.42 [20.67–22.19]), and sensorineural hearing loss (8.75 [7.85–9.77] vs. 5.13 [4.79–5.49]).

## 4 Discussion

This large, retrospective cohort study used a US EHR database to examine age‐, sex‐, and race‐matched patients ≥ 12 years with and without vitiligo. The study compared baseline comorbidities, surgical procedures, and history of prescription medication use prior to vitiligo diagnosis, as well as incidence of vitiligo based on age, sex, race, and ethnicity and IRs of safety events of interest following diagnosis. Autoimmune processes and oxidative stress play a role in the pathogenesis of vitiligo as well as other autoimmune diseases, such as autoimmune thyroid disorders, which are common in patients with vitiligo [9, 12, 13]. During the baseline period of the current study, autoimmune conditions including autoimmune thyroiditis, Hashimoto's thyroiditis, psoriasis, AA, atopic dermatitis, type 1 diabetes, Graves' disease, systemic lupus erythematosus, celiac disease, psoriatic arthritis, ulcerative colitis, myasthenia gravis, multiple sclerosis, and rheumatoid arthritis were more common among patients with vitiligo compared with matched controls. During the follow‐up period, patients with vitiligo had numerically higher IRs of AA, autoimmune blistering disease, autoimmune thyroiditis, bullous pemphigoid, myasthenia gravis, pernicious anemia, psoriasis, Sjogren's syndrome, systemic lupus erythematosus, systemic sclerosis, and type 1 diabetes than patients without vitiligo. These findings align with the pathogenesis of vitiligo and with previous studies, which have reported higher rates of autoimmune diseases in patients with vitiligo compared with controls, including thyroid disease (such as Hashimoto's thyroiditis and Graves' disease), psoriasis, AA, type 1 diabetes, systemic lupus erythematosus, myasthenia gravis, multiple sclerosis, rheumatoid arthritis, pernicious anemia, and Sjogren's syndrome [1, 9, 10, 11, 12, 13, 24, 25]. These findings build upon previous studies, providing further insight into the elevated risk of certain autoimmune conditions among the US patients with vitiligo, and may help inform more personalized treatment decisions and risk management strategies for these patients.

During the baseline period in our study, melanoma, NMSC, basal cell carcinoma, squamous cell carcinoma, and primary malignancy were more common in the vitiligo cohort than in the matched‐control cohort. Epstein–Barr–related lymphoma, Epstein–Barr–related leukemia, and lymphoma were also more common in the vitiligo cohort. The IRs of melanoma, NMSC, and primary malignancy after vitiligo diagnosis were also numerically higher in the vitiligo cohort compared with the non‐vitiligo cohort. However, these differences were small and previous studies have shown a decreased risk for melanoma, NMSC, and internal malignancies in patients with vitiligo [26, 27, 28, 29, 30]. Furthermore, a database study of primary care records in the UK of over 15 000 patients with vitiligo and 60 000 age‐ and sex‐matched controls found no difference in skin cancer prevalence at diagnosis and a 38% reduced risk of new‐onset skin cancer overall (median follow‐up, 7.7 years) among patients with vitiligo [27]. Similarly, a systematic review and meta‐analysis found the risk of basal cell carcinoma and squamous cell carcinoma was reduced in patients with vitiligo compared with healthy controls in multiple models [30]. In the current study, diagnostic codes alone were used to identify patients with skin cancer, and this method may not have accurately estimated skin cancer rates [31, 32, 33]. For example, a study that compared NMSC cases identified in claims data via *ICD* codes alone with cases found through natural language processing of electronic pathology reports found that *ICD* codes alone had sensitivities of 64% to 65% and specificities of 85% to 96%, which may not be sufficient for accurate case identification [32]. Studies have also found that *ICD* codes alone are not as accurate at identifying skin cancer cases compared with using *ICD* codes in combination with current procedural terminology codes [31, 33]. Supplementing the Optum EHR data with chart review or natural language processing of unstructured data, as well as external validation in other cohorts, is likely needed before drawing any conclusions from these data about the differences in malignancy rates between patients with and without vitiligo.

After vitiligo diagnosis, IRs of infections, including herpes simplex, herpes zoster, and opportunistic infection, were numerically higher among patients with vitiligo than those without vitiligo. Some of these infections, such as herpes simplex and herpes zoster, were also more prevalent at baseline in the vitiligo cohort. The higher use of immunosuppressive therapies among patients with vitiligo may partially explain the greater rates of infection. Treatments for vitiligo can include systemic corticosteroids or other immunosuppressants [14], which are known to increase susceptibility to infections. As oral targeted immunomodulators enter clinical development for vitiligo treatment [17, 18], this becomes particularly relevant. JAK inhibitors have been shown to be associated with risk of infection [21, 22], underscoring the importance of understanding infection risk in patients with vitiligo. These findings are critical for contextualizing safety signals in clinical trials and guiding treatment decisions in real‐world settings. In addition, future studies evaluating infection risk in patients with vitiligo that account for treatment patterns over time are warranted.

Additional conditions with numerically higher IRs among patients with vitiligo vs. those without during the follow‐up period included hearing loss, sensorineural hearing loss, and paresthesia or dysesthesia. At baseline, more patients in the vitiligo cohort also had hearing loss than did those in the non‐vitiligo cohort. An increased risk of auditory conditions in patients with vitiligo has been reported in other studies. These include findings pertaining to lower hearing thresholds of the inner ear [34] and increased odds of sensorineural hypoacusis (odds ratio [OR], 2.43; 95% CI, 1.50–3.93) and hypoacusis (OR, 1.29; 95% CI, 1.25–1.34) [9]. The authors of this study suggested that the observed higher rates of auditory conditions may be due to the involvement of melanocytes in the inner ear, which are thought to play a role in auditory function and may be affected in vitiligo.

In this study, IRs of psychiatric‐related comorbidities (depression, psychiatric conditions, and suicidal ideation) were lower in patients with vitiligo compared with matched controls during the follow‐up period, which is contrary to published literature [7, 35]. A systematic literature review of 168 studies examining psychosocial comorbidities in patients with vitiligo found that the prevalence of these conditions, especially depression and anxiety, was significantly higher among patients with vitiligo than controls [7]. Similarly, a global survey of 3541 patients with vitiligo found that 24.5% reported a formal diagnosis of depression and 55.0% had moderate to severe depression symptoms [35]. Several factors may explain the lower prevalence of depression or other psychiatric conditions in this particular vitiligo cohort compared with matched controls. Mental health conditions can be underreported due to various factors that affect whether patients seek care—including age, sex, employment, ethnicity, insurance coverage, disorder severity, and stigma associated with mental health conditions [36, 37, 38]. Additionally, the control group represents a healthcare‐seeking population and may have higher rates of psychiatric comorbidities compared with the general population. The vitiligo cohort was also older relative to the common age for vitiligo onset, which is often between 10 and 30 years of age [1]. Although disease duration could not be determined, these older patients may have had longer disease duration and thus developed coping mechanisms for psychosocial comorbidities over time, resulting in a lower prevalence of these psychiatric conditions. Consistent with this, prior studies have shown that younger age is significantly associated with psychosocial comorbidities in patients with vitiligo [7, 35]. In addition, in the US adult population, the prevalence of anxiety and depression is greater among younger adults and decreases with age [39]. The vitiligo cohort in this study also had a numerically lower baseline prevalence of psychiatric‐related comorbidities than the non‐vitiligo cohort, which may have influenced comparisons of psychiatric IRs during follow‐up.

For cardiovascular‐related comorbidities, lower IRs after vitiligo diagnosis were observed in patients with vitiligo compared with matched controls. At baseline, the overall prevalence of cardiovascular‐related conditions was generally comparable between cohorts, and a few cardiovascular conditions, including hypertension, coronary artery bypass graft or percutaneous coronary intervention, venous thromboembolism or arterial thromboembolism, and ischemic stroke, were slightly less prevalent in the vitiligo cohort. Previous studies of cardiovascular‐related diseases in patients with vitiligo have conflicting results, with some demonstrating increased risk for certain cardiovascular conditions in patients with vitiligo compared with controls, while others show comparable or decreased risk [40, 41, 42, 43]. Differences in study populations, methodologies, and other variables (treatment history and lifestyle factors) likely play a role, and further research is needed to clarify the risk of cardiovascular comorbidities in patients with vitiligo.

As expected, the history of prescription medication usage for dermatologic conditions was higher among patients with vitiligo than those without. Specifically, patients with vitiligo had a greater proportion of usage of systemic corticosteroids (including betamethasone, hydrocortisone, cortisone, prednisone, prednisolone, or triamcinolone), systemic steroids (including glucocorticoids), systemic immunosuppressants, topical calcineurin inhibitors, and topical corticosteroids. As the assessment window for history of prescription medication usage included 30 days after the index date, these results could include medications that were prescribed at vitiligo diagnosis. In addition, the higher proportion of patients with baseline autoimmune conditions in the vitiligo cohort could contribute to the increased use of immunosuppressive therapies, as these medications are commonly used to manage autoimmune diseases [44, 45].

Estimates of the incidence, prevalence, and cumulative incidence of vitiligo stratified by age, ethnicity, race, and sex revealed numerically higher rates among Asian and Hispanic populations. Although the vitiligo cohort included a relatively small proportion of Asian (843; 5.6%) and Hispanic patients (1706; 11.3%), which may limit the generalizability of these results, the proportion of Asian patients is comparable to that of the general US population, whereas the proportion of Hispanic patients is slightly lower [46] and these findings are consistent with prior studies. One US‐based study found that Asian American patients had the highest age‐adjusted IR (41.2 per 100 000 PY; 95% CI, 28.2–58.2), followed by Hispanic/Latino patients (37.3 per 100 000 PY; 95% CI, 25.7–52.4); however, these populations comprised a small proportion of that study's cohort (Asian American, 1.4%; Hispanic/Latino, 1.5%) [47]. Similarly, a UK study of vitiligo incidence found lifetime incidence was highest among Asian patients ([3.58%; 95% CI, 3.38–3.78]) compared with other racial and ethnic groups (Black, mixed/multiple, other, and White) [48]. In that study, the authors proposed that vitiligo may be more visible on darker skin tones. As a result, individuals in these population may be more likely to seek medical attention for vitiligo, which would contribute to higher reported incidence rates.

This study had several limitations inherent to the retrospective analysis of an EHR database. First, the higher rates of certain baseline comorbidities, such as melanoma, NMSC, and other dermatologic diseases among patients with vitiligo, may be reflective of ascertainment bias, given that the vitiligo cohort had substantially more dermatologist visits at baseline than the non‐vitiligo cohort (mean [SD], 1.8 [8.9] vs. 0.2 [1.4]). Second, differences between the present study and others may be attributable to the nature of the Optum EHR database, which captures only treated patients and uses structured diagnosis codes. Disease prevalence estimates derived from EHR databases have been shown to underestimate disease prevalence when using diagnostic codes alone, especially for diseases that are often undiagnosed or misdiagnosed, including skin cancer [31, 32, 33]. Third, the dataset did not capture the timing of vitiligo onset relative to comorbidities; thus, the temporal relationship between vitiligo onset and comorbid conditions could not be evaluated. Fourth, vitiligo status, demographics, treatment histories, comorbidities, and adverse events of interest may have been subject to misclassification. However, this risk was mitigated by requiring a diagnosis of vitiligo either from a dermatologist or from at least 2 separate vitiligo diagnoses, spaced at least 30 days apart, by a non‐dermatologist clinician. Fifth, although the vitiligo and non‐vitiligo cohorts were matched on age, sex, and race, residual confounding may still be present and could contribute to differences in observed outcome rates, independent of vitiligo status. Sixth, due to coding limitations, vitiligo subtypes are not identifiable using diagnostic codes alone in the Optum US EHR databases; therefore, it is not possible to distinguish between segmental vitiligo and nonsegmental vitiligo subtypes. Seventh, it is not possible to determine the specific condition for which a particular treatment was prescribed, and the index visit used in this study may not reflect a patient's initial vitiligo diagnosis. Eighth, because the Optum EHR database does not capture untreated patients and a larger proportion of patients were from the Midwest compared with the general US population in both cohorts [49], study results may not generalize to the entire US population. Ninth, as the study population reflects individuals who seek healthcare, findings may be subject to selection bias. Accordingly, the reported prevalence and incidence estimates likely reflect a healthcare‐seeking population and not the general population. Finally, as this study was based on a US dataset, the findings may not be applicable to populations in other countries. Despite these limitations, this study offers a comprehensive analysis of baseline comorbidities, history of surgical procedures and prescription medication use, IRRs for key outcome events, and the cumulative incidence of vitiligo in a large cohort of age‐, sex‐, and race‐ matched patients with and without vitiligo in the US.

## 5 Conclusions

This retrospective, observational cohort study examined baseline comorbidities, incidence and prevalence of vitiligo, and IRs of safety events of interest in a large cohort of patients with vitiligo compared with age‐, sex‐, and race‐matched controls. These findings revealed that certain autoimmune and inflammatory conditions, specific infections, select malignancies, hypothyroidism, allergic rhinitis, and hearing loss were more common at baseline among patients with vitiligo compared with those without. Asian populations in this US cohort had a numerically higher incidence and prevalence of vitiligo than other racial groups, as did Hispanic populations compared with non‐Hispanic populations. During the follow‐up period, higher IRs of several comorbidities were observed in the vitiligo cohort compared with the non‐vitiligo cohort, including autoimmune and inflammatory conditions, infections, and hearing‐related events, consistent with previous studies. Higher IRs of malignancies, including NMSC, were also observed, which contrasts with prior research and should be interpreted with caution considering the limitations of EHR database studies. These findings contribute to a more comprehensive understanding of the epidemiology of vitiligo in the US and may inform future clinical management and therapeutic decision‐making for this patient population.

## Ethics Statement

All records were deidentified in compliance with the Health Insurance Portability and Accountability Act, and thus neither obtaining informed consent from patients nor approval from an Institutional Review Board was required.

## Conflicts of Interest

K.C., R.A., A.L., M.A.G., S.P.K., T.L., E.N., S.K.K., and L.N. are employees of and own stock/stock options in Pfizer Inc. N.M.E. has served as a consultant, advisory board member, and/or speaker for Avita, Incyte, VisualDx, La Roche‐Posay, Beiersdorf, Allergan, Eli Lilly, Galderma, Pfizer, Takeda, AbbVie, Janssen, Sanofi, L'Oréal, McGraw Hill, Dior, Medscape, and Canfield. She has grant funding from Pfizer, has received royalties from McGraw Hill, and has stock options in VisualDx. T.E. is an analytic consultant for Pfizer Inc. I.H. is a consultant for AbbVie, Pfizer, Incyte, UCB, Boehringer Ingelheim, Sonoma, Merck, Union Therapeutics, Novartis, Jansen, Avita, Galderma, Vimela, and Almirall; an investigator for Lenicura, Pfizer, Incyte, Avita, L'Oréal/La Roche‐Posay, ITN, and AbbVie; and a board member and past president of the Hidradenitis Suppurativa Foundation and Global Vitiligo Foundation.

## Supporting information


**File S1:** Lists of *International Classification of Diseases (ICD) 9 or 10, Clinical Modification Procedure Classification System (PCS) (ICD‐9‐PCS/ICD‐10‐PCS)*, current procedural terminology (CPT), National Drug Center (NDC), and Healthcare Common Procedure Coding System (HCPCS) codes used to identify diagnoses, surgical procedures, and drug treatments.
**Table S1:** Variables and associated washout periods.

## Data Availability

The data that support the findings of this study are available from Optum and are not publicly available. Restrictions apply to the availability of these data, which were used under license for this study File S1.

## References

[1] C. Bergqvist and K. Ezzedine , “Vitiligo: A Review,” Dermatology 236, no. 6 (2020): 571–592, 10.1159/000506103. 32155629

[2] J. Akl , S. Lee , H. J. Ju , et al., “Estimating the Burden of Vitiligo: A Systematic Review and Modelling Study,” Lancet Public Health 9 (2024): e386–e396, 10.1016/S2468-2667(24)00026-4. 38552651

[3] Y. Yamaguchi and V. J. Hearing , “Melanocytes and Their Diseases,” Cold Spring Harbor Perspectives in Medicine 4, no. 5 (2014): a017046, 10.1101/cshperspect.a017046. 24789876 PMC3996377

[4] K. Ezzedine , V. Eleftheriadou , M. Whitton , and N. van Geel , “Vitiligo,” Lancet 386, no. 9988 (2015): 74–84, 10.1016/S0140-6736(14)60763-7. 25596811

[5] M. Picardo , M. L. Dell'Anna , K. Ezzedine , et al., “Vitiligo,” Nature Reviews. Disease Primers 1 (2015): 15011, 10.1038/nrdp.2015.11. 27189851

[6] A. Taïeb and M. Picardo , “Clinical Practice. Vitiligo,” New England Journal of Medicine 360, no. 2 (2009): 160–169, 10.1056/NEJMcp0804388. 19129529

[7] K. Ezzedine , V. Eleftheriadou , H. Jones , et al., “Psychosocial Effects of Vitiligo: A Systematic Literature Review,” American Journal of Clinical Dermatology 22, no. 6 (2021): 757–774, 10.1007/s40257-021-00631-6. 34554406 PMC8566637

[8] G. Wang , D. Qiu , H. Yang , and W. Liu , “The Prevalence and Odds of Depression in Patients With Vitiligo: A Meta‐Analysis,” Journal of the European Academy of Dermatology and Venereology 32, no. 8 (2018): 1343–1351, 10.1111/jdv.14739. 29222958

[9] J. H. Lee , H. J. Ju , J. M. Seo , et al., “Comorbidities in Patients With Vitiligo: A Systematic Review and Meta‐Analysis,” Journal of Investigative Dermatology 143, no. 5 (2023): 777–789.e6, 10.1016/j.jid.2022.10.021. 36574529

[10] K. Ezzedine , K. P. Anastassopoulos , K. Gandhi , et al., “A Survey Study of Self‐Reported Comorbidities Among Adults With Vitiligo in the United States,” JEADV Clinical Practice 2, no. 2 (2023): 300–305, 10.1002/jvc2.111.

[11] Z. Hu and T. Wang , “Beyond Skin White Spots: Vitiligo and Associated Comorbidities,” Frontiers in Medicine 10 (2023): 1072837, 10.3389/fmed.2023.1072837. 36910477 PMC9995999

[12] V. M. Sheth , Y. Guo , and A. A. Qureshi , “Comorbidities Associated With Vitiligo: A Ten‐Year Retrospective Study,” Dermatology 227, no. 4 (2013): 311–315, 10.1159/000354607. 24107643

[13] K.‐C. Fan , T.‐H. Yang , and Y.‐C. Huang , “Vitiligo and Thyroid Disease: A Systematic Review and Meta‐Analysis,” European Journal of Dermatology 28, no. 6 (2018): 750–763, 10.1684/ejd.2018.3449. 30698146

[14] M. Böhm , J. A. Schunter , K. Fritz , et al., “ *S*1 Guideline: Diagnosis and Therapy of Vitiligo,” Journal der Deutschen Dermatologischen Gesellschaft 20, no. 3 (2022): 365–378, 10.1111/ddg.14713. 35246935

[15] V. Eleftheriadou , R. Atkar , J. Batchelor , et al., “British Association of Dermatologists Guidelines for the Management of People With Vitiligo 2021,” British Journal of Dermatology 186, no. 1 (2022): 18–29, 10.1111/bjd.20596. 34160061

[16] D. Rosmarin , T. Passeron , A. G. Pandya , et al., “Two Phase 3, Randomized, Controlled Trials of Ruxolitinib Cream for Vitiligo,” New England Journal of Medicine 387, no. 16 (2022): 1445–1455, 10.1056/NEJMoa2118828. 36260792

[17] S. Inoue , T. Suzuki , S. Sano , and I. Katayama , “Jak Inhibitors for the Treatment of Vitiligo,” Journal of Dermatological Science 113, no. 3 (2024): 86–92, 10.1016/j.jdermsci.2023.12.008. 38326166

[18] K. Ezzedine , E. Peeva , Y. Yamaguchi , et al., “Efficacy and Safety of Oral Ritlecitinib for the Treatment of Active Nonsegmental Vitiligo: A Randomized Phase 2b Clinical Trial,” Journal of the American Academy of Dermatology 88, no. 2 (2023): 395–403, 10.1016/j.jaad.2022.11.005. 36370907

[19] T. Passeron , K. Ezzedine , I. Hamzavi , et al., “Once‐Daily Upadacitinib Versus Placebo in Adults With Extensive Non‐Segmental Vitiligo: A Phase 2, Multicentre, Randomised, Double‐Blind, Placebo‐Controlled, Dose‐Ranging Study,” EClinicalMedicine 73 (2024): 102655, 10.1016/j.eclinm.2024.102655. 38873632 PMC11169949

[20] A. G. Pandya , K. Ezzedine , T. Passeron , et al., “Efficacy and Safety of the Oral Janus Kinase 1 Inhibitor Povorcitinib in Patients With Extensive Vitiligo in a Phase 2, Randomized, Double‐Blinded, Dose‐Ranging, Placebo‐Controlled Study,” Journal of the American Academy of Dermatology 93 (2025): 946–955, 10.1016/j.jaad.2025.06.027. 40518122

[21] C. Samuel , H. Cornman , A. Kambala , and S. G. Kwatra , “A Review on the Safety of Using Jak Inhibitors in Dermatology: Clinical and Laboratory Monitoring,” Dermatol. Ther. (Heidelb.) 13 (2023): 1–21, 10.1007/s13555-023-00892-5. 36790724 PMC9930707

[22] F. Sunzini , I. McInnes , and S. Siebert , “Jak Inhibitors and Infections Risk: Focus on Herpes Zoster,” Therapeutic Advances in Musculoskeletal Disease 12 (2020): 1759720X20936059, 10.1177/1759720X20936059. PMC7328488 32655703

[23] A. S. Gershon , L. Warner , P. Cascagnette , J. C. Victor , and T. To , “Lifetime Risk of Developing Chronic Obstructive Pulmonary Disease: A Longitudinal Population Study,” Lancet 378, no. 9795 (2011): 991–996, 10.1016/s0140-6736(11)60990-2. 21907862

[24] J. M. Bae , J. H. Lee , J. S. Yun , B. Han , and T. Y. Han , “Vitiligo and Overt Thyroid Diseases: A Nationwide Population‐Based Study in Korea,” Journal of the American Academy of Dermatology 76, no. 5 (2017): 871–878, 10.1016/j.jaad.2016.12.034. 28238453

[25] Z. A. Abdel‐Malek , C. Jordan , T. Ho , P. R. Upadhyay , A. Fleischer , and I. Hamzavi , “The Enigma and Challenges of Vitiligo Pathophysiology and Treatment,” Pigment Cell & Melanoma Research 33, no. 6 (2020): 778–787, 10.1111/pcmr.12878. 32198977

[26] J. M. Bae , K. Y. Chung , S. J. Yun , et al., “Markedly Reduced Risk of Internal Malignancies in Patients With Vitiligo: A Nationwide Population‐Based Cohort Study,” Journal of Clinical Oncology 37, no. 11 (2019): 903–911, 10.1200/jco.18.01223. 30785828

[27] J. Ferguson , V. Eleftheriadou , and J. Nesnas , “Risk of Melanoma and Nonmelanoma Skin Cancer in People With Vitiligo: United Kingdom Population−Based Cohort Study,” Journal of Investigative Dermatology 143, no. 11 (2023): 2204–2210, 10.1016/j.jid.2023.04.013. 37146674

[28] H. E. Teulings , M. Overkamp , E. Ceylan , et al., “Decreased Risk of Melanoma and Nonmelanoma Skin Cancer in Patients With Vitiligo: A Survey Among 1307 Patients and Their Partners,” British Journal of Dermatology 168, no. 1 (2013): 162–171, 10.1111/bjd.12111. 23136900

[29] A. Paradisi , S. Tabolli , B. Didona , L. Sobrino , N. Russo , and D. Abeni , “Markedly Reduced Incidence of Melanoma and Nonmelanoma Skin Cancer in a Nonconcurrent Cohort of 10,040 Patients With Vitiligo,” Journal of the American Academy of Dermatology 71, no. 6 (2014): 1110–1116, 10.1016/j.jaad.2014.07.050. 25242557

[30] A. Rooker , W. Ouwerkerk , M. W. Bekkenk , R. M. Luiten , and W. J. Bakker , “The Risk of Keratinocyte Cancer in Vitiligo and the Potential Mechanisms Involved,” Journal of Investigative Dermatology 144, no. 2 (2024): 234–242, 10.1016/j.jid.2023.08.012. 37791932

[31] M. J. Eide , R. Krajenta , D. Johnson , et al., “Identification of Patients With Nonmelanoma Skin Cancer Using Health Maintenance Organization Claims Data,” American Journal of Epidemiology 171, no. 1 (2010): 123–128, 10.1093/aje/kwp352. 19969529 PMC2796985

[32] M. J. Eide , J. Mark Tuthill , R. J. Krajenta , G. R. Jacobsen , M. Levine , and C. C. Johnson , “Validation of Claims Data Algorithms to Identify Nonmelanoma Skin Cancer,” Journal of Investigative Dermatology 132, no. 8 (2012): 2005–2009, 10.1038/jid.2012.98. 22475754 PMC3393824

[33] N. Anand , L. Edwards , L. X. Baker , M.‐M. Chren , and L. Wheless , “Validity of Using Billing Codes From Electronic Health Records to Estimate Skin Cancer Counts,” JAMA Dermatology 157, no. 9 (2021): 1089–1094, 10.1001/jamadermatol.2021.2856. 34379079 PMC8358802

[34] K. F. A. E. Gaml , H. A. K. Mohamed , R. M. Eldahshan , T. A. Gabr , and M. L. Elsaie , “Assessment of Auditory and Cochlear Function in Non Segmental Vitiligo Patients: A Case Controlled Study,” Archives of Dermatological Research 317, no. 1 (2025): 409, 10.1007/s00403-025-03912-w. 39951129

[35] K. Ezzedine , D. Parsad , J. E. Harris , et al., “Depression and Depressive Symptoms Among People Living With Vitiligo: Findings From the Cross‐Sectional, Population‐Based Global Valiant Survey,” Journal of Dermatological Treatment 36, no. 1 (2025): 2504082, 10.1080/09546634.2025.2504082. 40464715

[36] M. G. Harris , A. E. Kazdin , I. Hwang , et al., “Pathway to Effective Treatment for Common Mental and Substance Use Disorders in the World Mental Health Surveys: Perceived Need for Treatment,” International Journal of Mental Health Systems 19, no. 1 (2025): 17, 10.1186/s13033-025-00666-w. 40410765 PMC12100881

[37] J. L. Magaard , T. Seeralan , H. Schulz , and A. L. Brütt , “Factors Associated With Help‐Seeking Behaviour Among Individuals With Major Depression: A Systematic Review,” PLoS One 12, no. 5 (2017): e0176730, 10.1371/journal.pone.0176730. 28493904 PMC5426609

[38] G. Schomerus , S. Stolzenburg , S. Freitag , et al., “Stigma as a Barrier to Recognizing Personal Mental Illness and Seeking Help: A Prospective Study Among Untreated Persons With Mental Illness,” European Archives of Psychiatry and Clinical Neuroscience 269, no. 4 (2019): 469–479, 10.1007/s00406-018-0896-0. 29679153

[39] E. P. Terlizzi and B. Zablotsky , “Symptoms of Anxiety and Depression Among Adults: United States, 2019 and 2022,” National Center for Health Statistics, National Health Statistics Report 213 (2024): CS353885, https://stacks.cdc.gov/view/cdc/164018. 10.15620/cdc/64018 PMC11616099 39591466

[40] S. M. Rahman , M. Wang , F. Ahmed , and M. Jafferany , “Assessing the Relationship Between Vitiligo and Cardiovascular Disease Risk Factors,” European Medical Journal (2023): 10308019, 10.33590/emj/10308019.

[41] K. Kridin , N. Kafri , and A. D. Cohen , “Is Vitiligo Associated With an Increased Risk of Cardiovascular Outcomes? Perceptions From a Population‐Based Study,” Australasian Journal of Dermatology 65, no. 4 (2024): 384–386, 10.1111/ajd.14251. 38528737

[42] A. Frączek , A. Owczarczyk‐Saczonek , R. J. Ludwig , et al., “Vitiligo Is Associated With an Increased Risk of Cardiovascular Diseases: A Large‐Scale, Propensity‐Matched, US‐Based Retrospective Study,” eBioMedicine 109 (2024): 105423, 10.1016/j.ebiom.2024.105423. 39461193 PMC11543909

[43] X. Liang , F. Guo , and M. Zhang , “Risk Factors for Cardiovascular Diseases in Patients With Vitiligo: An Analysis of Current Evidence,” Annals of Medicine 56, no. 1 (2024): 2326297, 10.1080/07853890.2024.2326297. 39300810 PMC11418058

[44] D. A. Lintzeri , A. Constantinou , K. Hillmann , K. Ghoreschi , A. Vogt , and U. Blume‐Peytavi , “Alopecia Areata ‐ Current Understanding and Management,” Journal der Deutschen Dermatologischen Gesellschaft 20, no. 1 (2022): 59–90, 10.1111/ddg.14689. 35040577

[45] L. C. Coates , E. R. Soriano , N. Corp , et al., “Group for Research and Assessment of Psoriasis and Psoriatic Arthritis (Grappa): Updated Treatment Recommendations for Psoriatic Arthritis 2021,” Nature Reviews Rheumatology 18, no. 8 (2022): 465–479, 10.1038/s41584-022-00798-0. 35761070 PMC9244095

[46] KFF , “Population Distribution by Race/Ethnicity,” accessed March 30, 2026, Population Distribution by Race/Ethnicity.

[47] N. Mastacouris , A. Strunk , and A. Garg , “Incidence and Prevalence of Diagnosed Vitiligo According to Race and Ethnicity, Age, and Sex in the US,” JAMA Dermatology 159, no. 9 (2023): 986–990, 10.1001/jamadermatol.2023.2162. 37466934 PMC10357354

[48] V. Eleftheriadou , A. Ahmed , J. Nesnas , and R. Nagra , “The Lifetime Risk and Impact of Vitiligo Across Sociodemographic Groups: A UK Population‐Based Cohort Study,” British Journal of Dermatology 192, no. 1 (2024): 63–71, 10.1093/bjd/ljae282. 39018020

[49] V. Simpson , How Is Population Distributed Across the United States World Atlas, accessed March 26, 2025, https://www.worldatlas.com/articles/how‐is‐population‐distributed‐across‐the‐united‐states.html.

