# Supplementary material for: Risk of Safety Events in Vitiligo Patients: A Retrospective Real‐World Data Study in the US
Source: J Dermatol. 2026 Apr 6;53(5):758–73. doi: 10.1111/1346-8138.70256 (PMC13150671; doi:10.1111/1346-8138.70256)
Supplement: Supplementary file 1 — File S1: Lists of International Classification of Diseases (ICD) 9 or 10, Clinical Modification Procedure Classification System (PCS) (ICD‐9‐PCS/ICD‐10‐PCS), current procedural terminology (CPT), National Drug Center (NDC), and Healthcare Common Procedure Coding System (HCPCS) codes used to identify diagnoses, surgical procedures, and drug treatments. Table S1: Variables and associated washout periods. [file JDE-53-758-s001.docx]

**SUPPORTING INFORMATION**

**Risk of safety events in vitiligo patients: a retrospective real-world data study in the US**

Kennedy Cook,^1^ Nada M. Elbuluk,^2^ Roni Adiri,^3^ Alexandre Lejeune,^4^ Thomas Edwards,^5^ Milena A. Gianfrancesco,^1^ Scott P. Kelly,^1^ Tatjana Lukic,^1^ Edward Nagy,^6^ Samantha K. Kurosky,^1^ Lynne Napatalung,^1,7^ Iltefat Hamzavi^8^

^1^Pfizer Inc, New York, New York, USA; ^2^Department of Dermatology, Keck School of Medicine, University of Southern California, Los Angeles, California, USA ^3^Pfizer Pharmaceuticals Israel Ltd., Herzliya, Israel; ^4^Pfizer Inc, Paris, France; ^5^Panalgo, LLC, Westland, Michigan, USA; ^6^Pfizer Inc, Collegeville, Pennsylvania, USA; ^7^Department of Dermatology, Icahn School of Medicine at Mount Sinai, New York, NY, USA; ^8^Henry Ford Health System, Department of Dermatology, Detroit, Michigan, USA

**Supplementary File S1**. Lists of *International Classification of Diseases (ICD) 9 or 10, Clinical Modification Procedure Classification System (PCS) (ICD-9-PCS/ICD-10-PCS)*, current procedural terminology (CPT), National Drug Center (NDC), and Healthcare Common Procedure Coding System (HCPCS) codes used to identify diagnoses, surgical procedures, and drug treatments

**Table S1.** Variables and associated washout periods

| **Variable** | **Assessment window** | **Washout** |
| --- | --- | --- |
| **Comorbidities** |  |  |
| Allergic rhinitis | Baseline period plus 30 days after index date | N/A |
| Alopecia areata | Baseline period plus 30 days after index date |  |
| Asthma | Baseline period plus 30 days after index date |  |
| Atopic dermatitis | Baseline period plus 30 days after index date |  |
| Diabetes | Baseline period plus 30 days after index date |  |
| Dyslipidemia | Baseline period plus 30 days after index date |  |
| Hyperlipidemia | Baseline period plus 30 days after index date |  |
| EBV-related lymphoproliferative disorder | Baseline period plus 30 days after index date |  |
| Hypertension | Baseline period plus 30 days after index date |  |
| Hypothyroidism | Baseline period plus 30 days after index date |  |
| Myasthenia gravis | Baseline period plus 30 days after index date |  |
| Autoimmune thyroiditis | Baseline period plus 30 days after index date |  |
| Hashimoto’s thyroiditis | Baseline period plus 30 days after index date |  |
| Grave’s disease | Baseline period plus 30 days after index date |  |
| Herpes zoster | Baseline period plus 30 days after index date |  |
| Herpes simplex | Baseline period plus 30 days after index date |  |
| HIV infection | Baseline period plus 30 days after index date |  |
| Hepatitis B infection | Baseline period plus 30 days after index date |  |
| Hepatitis C infection | Baseline period plus 30 days after index date |  |
| Primary malignancy (excluding NMSC) | Baseline period plus 30 days after index date |  |
| NMSC | Baseline period plus 30 days after index date |  |
| BCC | Baseline period plus 30 days after index date |  |
| SCC | Baseline period plus 30 days after index date |  |
| Melanoma | Baseline period plus 30 days after index date |  |
| Cervical cancer in situ | Baseline period plus 30 days after index date |  |
| Breast cancer | Baseline period plus 30 days after index date |  |
| Lymphoma | Baseline period plus 30 days after index date |  |
| Leukemia | Baseline period plus 30 days after index date |  |
| EBV-related lymphoma | Baseline period plus 30 days after index date |  |
| EBV-related leukemia | Baseline period plus 30 days after index date |  |
| Acute myocardial infarction | Baseline period plus 30 days after index date |  |
| Unstable angina | Baseline period plus 30 days after index date |  |
| Ischemic stroke | Baseline period plus 30 days after index date |  |
| Hemorrhagic stroke | Baseline period plus 30 days after index date |  |
| Coronary revascularization by PCI or CABG | Baseline period plus 30 days after index date |  |
| VTE (DVT or PE) | Baseline period plus 30 days after index date |  |
| DVT | Baseline period plus 30 days after index date |  |
| PE | Baseline period plus 30 days after index date |  |
| ATE | Baseline period plus 30 days after index date |  |
| VTE or ATE | Baseline period plus 30 days after index date |  |
| Sensorineural hearing loss | Baseline period plus 30 days after index date |  |
| Anxiety disorders | Baseline period plus 30 days after index date |  |
| Mood disorders | Baseline period plus 30 days after index date |  |
| Personality disorders | Baseline period plus 30 days after index date |  |
| Suicidal ideation/behavior | Baseline period plus 30 days after index date |  |
| Any substance use disorder | Baseline period plus 30 days after index date |  |
| SLE | Baseline period plus 30 days after index date |  |
| Celiac disease | Baseline period plus 30 days after index date |  |
| Crohn’s disease | Baseline period plus 30 days after index date |  |
| Ulcerative colitis | Baseline period plus 30 days after index date |  |
| COPD | Baseline period plus 30 days after index date |  |
| Diabetes mellitus – type I | Baseline period plus 30 days after index date |  |
| Diabetes mellitus – type II | Baseline period plus 30 days after index date |  |
| Multiple sclerosis | Baseline period plus 30 days after index date |  |
| Psoriasis | Baseline period plus 30 days after index date |  |
| Psoriatic arthritis | Baseline period plus 30 days after index date |  |
| Rheumatoid arthritis | Baseline period plus 30 days after index date |  |
| Serious infection requiring hospitalization | Baseline period plus 30 days after index date |  |
| **Medications** |  |  |
| JAKi | Baseline period plus 30 days after index date; tofacitinib, baricitinib, upadacitinib, filgotinib, ruxolitinib | N/A |
| Drugs used in dermatology | Baseline period plus 30 days after index date; alefacept, certolizumab, etanercept, adalimumab, infliximab, golimumab, ustekinumab, apremilast |  |
| Systemic steroids/glucosteroids | Baseline period plus 30 days after index date; ATC H02, ATC H02AB (excluding H02AB12, H02AB14, H02AB15, H02AB17), including systemic glucocorticoids |  |
| Immunosuppressants | Baseline period plus 30 days after index date; ATC L04 |  |
| Antidiabetic agents | Baseline period plus 30 days after index date; ATC A10 |  |
| Lipid modifying agents | Baseline period plus 30 days after index date; ATC C10 |  |
| Antihypertensive agents | Baseline period plus 30 days after index date; ATC C02, C03, C07, C08, C09 |  |
| Hormone replacement therapy | Baseline period plus 30 days after index date; ATC G03F |  |
| Oral contraceptives | Baseline period plus 30 days after index date; (ATC G03F [progestins and estrogens] and G03CA57 and G03CC07 [conjugated estrogens] and G03HB01 [cyproterone and estrogen]), oral contraceptives (ATC G03A) |  |
| Non–B-cell-selective lymphocyte-depleting agents | Baseline period plus 30 days after index date; alemtuzumab |  |
| B-cell–selective lymphocyte-depleting agents | Baseline period plus 30 days after index date; rituximab and others |  |
| Other immunomodulatory agents | Baseline period plus 30 days after index date; alefacept, certolizumab, etanercept, adalimumab, anakinra, abatacept, infliximab, golimumab, ustekinumab, apremilast, secukinumab, ixekizumab, brodalumab, bimekizumab, guselkumab, risankizumab, tildrakizumab |  |
| Systemic immune suppressants | Baseline period plus 30 days after index date; cyclosporine A, tacrolimus, azathioprine, methotrexate, sulfasalazine, mycophenolate mofetil, everolimus, ibrutinib |  |
| Systemic corticosteroids (oral, injectable [intramuscular, intravenous]) | Baseline period plus 30 days after index date; betamethasone, hydrocortisone, cortisone, prednisone, prednisolone, triamcinolone |  |
| Intralesional corticosteroids | Baseline period plus 30 days after index date; betamethasone, hydrocortisone, cortisone, prednisone, prednisolone, triamcinolone |  |
| Topical corticosteroids | Baseline period plus 30 days after index date; betamethasone, hydrocortisone, cortisone, prednisone, prednisolone, triamcinolone |  |
| Topical vitamin D3 | Baseline period plus 30 days after index date |  |
| Topical calcineurin inhibitors | Baseline period plus 30 days after index date |  |
| Phototherapy | Baseline period plus 30 days after index date, e.g., excimer, broadband UVB, UVA, etc. |  |
| PUVA | Baseline period plus 30 days after index date |  |
| Laser | Baseline period plus 30 days after index date |  |
| Zoster vaccine live (Zostavax) | Baseline period plus 30 days after index date |  |
| Zoster vaccine recombinant (Shingrix) | Baseline period plus 30 days after index date |  |
| **Procedure** |  |  |
| Hysterectomy | Any history | N/A |
| Surgical treatments and camouflage | Baseline period plus 30 days after index date |  |
| Salpingectomy | Any history |  |
| Oophorectomy | Any history |  |
| **Outcomes** |  |  |
| Opportunistic infections | ≥30 days post-index | Acute, no washout |
| Herpes zoster | ≥30 days post-index | Washout for entire database availability pre-index [−∞, index date −1] |
| Herpes simplex | ≥30 days post-index |  |
| All cause death | ≥30 days post-index | Acute, no washout |
| Psychiatric conditions | ≥30 days post-index; anxiety, mood, and personality disorders, and suicidal ideation or behavior | Washout for entire database availability pre-index [−∞, index date −1] |
| Suicidal ideation | ≥30 days post-index | Acute, no washout |
| Depression | ≥30 days post-index | Washout for entire database availability pre-index [−∞, index date −1] |
| Diabetes (type 1 or type 2) | ≥30 days post-index |  |
| Type 1 diabetes | ≥30 days post-index |  |
| Type 2 diabetes | ≥30 days post-index |  |
| Autoimmune thyroiditis | ≥30 days post-index includes Hashimoto’s thyroiditis) |  |
| Rheumatoid arthritis | ≥30 days post-index |  |
| Systematic lupus erythematosus | ≥30 days post-index |  |
| Sjogren’s syndrome | ≥30 days post-index |  |
| Myasthenia gravis | ≥30 days post-index |  |
| Systemic sclerosis | ≥30 days post-index |  |
| Autoimmune blistering diseases (epidermolysis bullosa, pemphigus vulgaris, or bullous pemphigoid) | ≥30 days post-index |  |
| Epidermolysis bullosa | ≥30 days post-index |  |
| Pemphigus vulgaris | ≥30 days post-index |  |
| Bullous pemphigoid | ≥30 days post-index |  |
| Psoriasis | ≥30 days post-index |  |
| Pernicious anemia | ≥30 days post-index |  |
| Hearing loss | ≥30 days post-index |  |
| Alopecia areata | ≥30 days post-index |  |
| Serious infections | ≥30 days post-index | Acute, no washout. |
| Primary malignancy | ≥30 days post-index; excluding NMSC | Washout for entire database availability pre-index [−∞, index date −1] |
| NMSC | ≥30 days post-index, Includes BCC and SCC | Acute, no washout |
| DVT | ≥30 days post-index |  |
| PE | ≥30 days post-index |  |
| DVT or PE | ≥30 days post-index |  |
| VTE (DVT and PE combined) | ≥30 days post-index |  |
| Arterial thrombosis/ thromboembolism | ≥30 days post-index |  |
| DVT, PE, or arterial thrombosis/thromboembolism | ≥30 days post-index |  |
| ***MACE*** | ≥30 days post-index  Defined as a composite measure comprised of cardiovascular death, AMI, unstable angina, coronary revascularization, ischemic or hemorrhagic stroke, and heart failure with hospitalization. | Both acute and washout for entire database availability pre-index [−∞, index date −1] |
| Cardiovascular death | ≥30 days post-index  Defined as patient with any of following AND death occurring within 30 days after diagnosis/procedure:  AMI, sudden cardiac event, heart failure, stroke, cardiovascular procedures (including PTCA/PCI), cardiac transplant, CABG, coronary revascularization procedures (any catheter-based or open surgical procedure to improve myocardial blood flow), other cardiovascular causes (i.e., peripheral artery disease). | Acute, no washout |
| Acute myocardial infarction | ≥30 days post-index |  |
| Unstable angina | ≥30 days post-index | Washout for entire database availability pre-index [−∞, index date −1] |
| Coronary revascularization by PCI or CABG | ≥30 days post-index | Acute, no washout |
| Ischemic stroke | ≥30 days post-index |  |
| Hemorrhagic stroke | ≥30 days post-index |  |
| Heart failure with hospitalization | ≥30 days post-index; any hospitalization with heart failure as a primary diagnostic code |  |
| Peripheral neuropathy | ≥30 days post-index | Washout for entire database availability pre- index [−∞, index date −1] |
| Sensorineural hearing loss | ≥30 days post-index |  |
| Paresthesia and dysesthesia | ≥30 days post-index |  |

AMI, acute myocardial infarction; ATE, arterial thromboembolism; BCC, basal cell carcinoma; CABG, coronary artery bypass graft; COPD, chronic obstructive pulmonary disease; DVT, deep vein thromboembolism; EBV, Epstein-Barr virus; JAKi, Janus kinase inhibitor; MACE, major adverse cardiovascular event; N/A, not applicable; NMSC, non-melanoma skin cancer; PCI, percutaneous coronary intervention; PE, pulmonary embolism; PTCA, percutaneous transluminal coronary angioplasty; PUVA, psoralen plus UVA light; SCC, squamous cell carcinoma; SLE, systemic lupus erythematosus; UVA, ultraviolet A; UVB, ultraviolet B; VTE, venous thromboembolism.
